# Downregulation of NCL attenuates tumor formation and growth in HeLa cells by targeting the PI3K/AKT pathway

**DOI:** 10.1002/cam4.4569

**Published:** 2022-02-06

**Authors:** Jun Ying, Ruowang Pan, Zhouhao Tang, Jiayin Zhu, Ping Ren, Yang Lou, Enyong Zhang, Dadao Huang, Penghong Hu, Dong Li, Qiyu Bao, Peizhen Li

**Affiliations:** ^1^ School of Laboratory Medicine and Life Science/Institute of Biomedical Informatics Wenzhou Medical University Wenzhou China; ^2^ School of Forensic Medicine Xi'an Jiaotong University Xi'an China; ^3^ No. 906 Hospital of Joint Logistic Support Force of PLA Wenzhou China; ^4^ Wenzhou Medical University Renji College Wenzhou China; ^5^ Laboratory Animal Center Wenzhou Medical University Wenzhou China

**Keywords:** apoptosis, HeLa cells, molecular mechanism, nucleolin, PI3K/AKT pathway, RNA‐Seq

## Abstract

**Background:**

Nucleolin (NCL, C23) is a multifunctional phosphoprotein that plays a vital role in modulating the survival, proliferationand apoptosis of cancer cells. However, the effects of NCL on cervical cancer and the underlying mechanisms behind this are poorly understood.

**Methods:**

Lentiviral transfection technology was used to construct NCL knockdown cell lines. MTT, colony formation assays, and tumorigenic assays in vivo were performed to observe cell proliferation. HOECHST 33342 staining, flow cytometry, and caspase activity assay were used to test cell apoptosis. RNA‐Seq, Western blotting, and RT‐PCR were conducted to investigate the specific molecular mechanism.

**Results:**

NCL knockdown inhibited cell proliferation and promoted apoptosis both *in vivo* and *in vitro*. Mechanistic studies revealed that NCL knockdown inhibited the PI3K/AKT pathway by upregulating FGF, ITGA, TNXB, VEGF, Caspase 3, and Bax, as well as by downregulating AKT, GNB4, CDK6, IL6R, LAMA, PDGFD, PPP2RSA and BCL‐2. In addition, the expression levels of apoptosis‐related genes after using a PI3K inhibitor LY294002 were consistent with shRNA studies, while treatment with a 740Y‐P agonist showed the opposite effect.

**Conclusions:**

Our findings indicate that downregulation of NCL may be a novel treatment strategy forcervical cancer.

## Graphical abstract

Nucleolin (NCL) knockdown inhibited cell proliferation and promoted apoptosis both in vivo and in vitro. Mechanistic studies revealed that NCL knockdown inhibited the PI3K/AKT pathway by upregulating FGF, ITGA, TNXB, as well as by downregulating AKT, GNB4, CDK6, IL6R, LAMA, PDGFD, PPP2RSA and BCL‐2. In addition, the expression levels of apoptosis‐related genes after using a PI3K inhibitor LY294002 were consistent with shRNA studies. Downregulation of NCL may be a novel treatment strategy for cervical cancer.

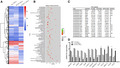

## 1 INTRODUCTION

Cervical cancer is a common malignant tumor of the female reproductive system with an increasing incidence.^1^ There are about 500,000 new cervical cancer cases worldwide each year, of which more than 130,000 cases are diagnosed in China.^2^ According to the World Health Organization (WHO), about 1 million patients worldwide will die of cervical cancer each year by the year 2050 based on the projected rate. In China, 20,000–30,000 people die from cervical cancer each year, even at a younger age. In addition, the 5‐year survival rate for cervical cancer is only 45.4%.^3^ Despite recent advances in cancer diagnosis and therapy, the prognosis and quality of life of patients are still not optimistic. Therefore, discovering new molecular targets and markers, increasing the survival rate, improving the prognosis and curative effects, and achieving precise treatment need to be the priority of research in the future.

In our previous work, the expression levels of nucleolin (NCL) in cervical cancer cells were shown to be downregulated after treatment with phycocyanin as shown by two‐dimensional gel electrophoresis (2DE) and mass spectrometry (MALDI‐TOF/MS), suggesting that NCL plays an important role in the development, cell proliferation, and apoptosis pathways. Studies have shown that NCL exhibits multiple biological functions, including regulation of cell proliferation and growth, embryogenesis, cytokinesis, chromatin replication, and the occurrence of the nucleolus and other processes.^4^, ^5^ It plays a regulatory role by directly binding to related proteins during DNA replication, recombination, and repair. Interestingly, NCL has recently been identified as apoptosis‐related proteins. Otake et al. found that Bcl‐2 protein levels increased 11‐fold when NCL increased 26‐fold in B‐lymphocytic leukemia.^6^ The expression levels of 110 kDa NCL in the nucleus reduced but an 80 kDa NCL degradation fragment appeared in human salivary gland cells, human oral squamous cell carcinoma cells (SCC‐25), and human osteoblasts (Saos‐2 and MG63) when induced by okadaic acid (OA).^7^, ^8^ In addition, Sanjeev et al. conducted miRNA transcriptomics analysis using HeLa cells depleted in NCL and found that the downregulation of NCL was associated with a decrease in cholesterol levels and an increase in fatty acid content, which was mainly due to the decreased and mislocalized expression of the transcription factor SREBP1 and the downregulation of enzymes involved in β‐oxidation and degradation of fatty acids.^9^ The expression levels of NCL are relatively high in tumors and other rapidly dividing cells but very low in nondividing cells. Using this feature as an effective indicator, NCL may be used to measure the degree of tumor cell proliferation and to judge sensitivity to certain drugs.

Even though there is accumulating evidence proving the pro‐tumor activity of NCL, data are still limited and the potential molecular mechanisms remain unknown. A treatment strategy using NCL as an entry point will bring hope to personalized treatment of diseases, especially tumors. In this study, we systematically investigated the role and underlying mechanisms of NCL knockdown in relation to cell growth both in vitro and in vivo to provide more detailed theoretical data and an experimental basis for the functional mechanisms of NCL and the diagnosis and treatment of cervical cancer.

## 2 MATERIALS AND METHODS

### 2.1 Cells

Human HeLa cervical cancer cells were obtained from the Cell Bank of the Chinese Academy of Sciences and cultured in DMEM (Thermo Fisher Scientific) supplemented with 10% fetal bovine serum (FBS; Sijiqing), 2 mM L‐glutamine, and antibiotics (100 U/ml streptomycins and 100 U/ml penicillin). Cells were maintained in a 37°C humidified 5% CO_2_ incubator.

### 2.2 Lentivirus constructs and transfections

Cells were designated into a blank control group (NC‐HeLa, uninfected HeLa cells), a negative control group (sh‐NT‐HeLa, infected with negative control vector, target sequence: 5′‐TTCTCCGAACGTGTCACGT‐3′), or an NCL (NM_005381.2) experimental group (sh‐NCL‐HeLa, infected with NCL knockdown vector, nucleolin‐RNAi [59416‐1], target sequence: 5′‐GGAAATGTCAGAAGATGAAGA‐3′). The RNAi lentiviral vector packaging system was constructed by Genechem. HeLa cells were seeded into six‐well plates at a density of 1 × 10^5^ per well. The next day, lentiviral vectors were transfected into HeLa cells using polybrene according to the manufacturer’s instructions. After transfection for 10 h, fresh DMEM was added and cultured for 48 h. Puromycin (4 μg/ml) was added to select stably transfected cells. This process was repeated three to four times until all cells expressed green fluorescent protein. Infection efficiency was determined using Western blotting assays.^10^

### 2.3 RNA isolation and quantification

Total RNA in different treatment groups was obtained using TRIzol® reagent (Invitrogen^™^) according to instructions provided by the manufacturer, and 2 μg aliquots were reversed transcribed cDNAs using PrimeScript^™^ RT‐PCR kit (Takara). After, cDNAs were subjected to PCR analyses using specific primers (the primer sequences are listed in Table 1) on an amplification detection system (Bio‐Rad). β‐actin served as a housekeeping gene. Messenger RNA (mRNA) expression levels were expressed as the ratio of the gray level of each sample to its internal reference β‐actin control and analyzed by the QuantityOne program (Bio‐Rad).^11^

**TABLE 1:** Primer sequences and product sizes

Gene	Primer sequences (5’‐3’)	Product size (bp)	Temp (°C)
*NCL*	F: CCACTTGTCCGCTTCACAC	352	55
	R: ACCAGGAGTTGCTACCAATG		
*PIK3R1*	F: TGTCAGTGATTGAAGAGCAT	348	55
	R: TACAGTCCAGAAGTTCCATAG		
*AKT*	F: GTATCTCAGTCTAAGGTCTCAT	448	60
	R: GGCTTCTTCTACAGTATCCA		
*GNB4*	F: TGGTGCTTGTGATGCCTCTT	378	55
	R: ACAGCCATGCCATCATCAGT		
*CDK6*	F: GTGAACCAGCCCAAGATGAC	116	60
	R: TGGAGGAAGATGGAGAGCAC		
*IL6R*	F: ACCAGACAGGTGCGAAAG	215	50
	R: CTCCCAAAGTGCTAGGATTA		
*LAMA1*	F: CTGACGGGTTCTATGGG	368	54
	R: GTCGCAGGTATTCTGAGTG		
*PDGFD*	F: AAGGAAACGGCTACG	290	50
	R: GTCATCGGACTTGAATG		
*PPP2R3A*	F: GCATTCCGACCTTCTACTT	292	55
	R: AGCAGATGGACGAACTTG		
*FGF2*	F: CCATTGTCCCAGTAAA	448	50
	R: CACCAGCAGAGTTGC		
*ITGA5*	F: GGGAGGTTTAGGAAGCG	251	58
	R: CCACTGAGAATCCGAAGAA		
*PRKCA*	F: TAGCACCGTCCGAATC	331	50
	R: TGAAAGGAAGGGAAGCA		
*TNXB*	F: TGGGAAGGCTACGTGAGTG	340	58
	R: CTGGGCATGTCTGGATGG		
*VEGF*	F: CTTGCCTTGCTGCTCTAC	278	58
	R: ATGGTGATGTTGGACTCCT		
*β‐actin*	F: CTACAATGAGCTGCGTGTGG	528	58
	R: AAGGAAGGCTGGAAGAGTGC		

Abbreviation: NCL, nucleolin.

### 2.4 Western blotting analysis

Protein was extracted from cells using RIPA lysis buffer, and protein levels were measured using a BCA kit (Beyotime). Then, 30 μg protein samples were separated using a 10% SDS‐PAGE and transferred to a PVDF membrane. Next, the membrane was washed using 5% skimmed milk diluted in TBST for 2 h, followed by incubation with primary antibodies at 4°C overnight. After being washed with TBST (containing 0.1% Tween‐20), the membranes were incubated with corresponding secondary antibodies for 2 h at room temperature. Immune complexes were examined using ECL detection (Beyo ECL Plus; Beyotime). Protein bands were quantified using ImageJ software. The primary antibodies used in Western blotting included: anti‐NCL (no. 10556), anti‐PI3K (no. 60225), anti‐AKT (no. 60203), anti‐phospho‐PI3K (no. AF3241), anti‐phospho‐AKT (no. 66444), anti‐Bax (no. 50599), anticleaved‐Caspase‐3 (no. AF7022), anti‐Bcl‐2 (no. 12789), and anti‐β‐Actin (no. 20536). These antibodies were purchased from Proteintech Group, Inc.^12^

### 2.5 Cell viability assay

Cells (1000 per well) were seeded into 96‐well plates and incubated at 37°C for 1–6 days. Next, 3‐(4, 5‐dimethylthiazol‐2‐yl)‐2, 5‐diphenyltetrazolium bromide (MTT) was added to each well at a final concentration of 0.5 mg/ml for 4 h. The supernatants were transferred, dimethyl sulfoxide was added, and the absorbance of each well was measured at 490 nm using an ELISA microplate reader (Bio‐Rad).^13^

### 2.6 Colony formation assays

Cells (density of 5 × 10^2^ per well) were reseeded into six‐well plates and incubated for 7–14 days at 37°C, with the medium changed every 3 days. Cells were fixed in 100% methanol for 15 min at 4°C and then stained with crystal violet (1%, wt/vol; Beyotime) for 15 min. An inverted microscope was used to count the number of newly formed colonies >200 μm in diameter.^14^

### 2.7 HOECHST 33342 staining

Cells in each group which were cultured for 48 h after subculture were washed twice with PBS. Next, 1 ml of 5 μg/ml Hoechst 33342 (Beyotime) working solution was added, and cells were incubated for 30 min at 37°C and then were observed and photographed using an inverted fluorescence microscope.^15^

### 2.8 Detection of apoptosis by FCM

Annexin V‐APC/7AAD Apoptosis Detection kit (BD Biosciences) was used to measure apoptosis levels, following manufacturer instructions. Cells were collected, washed twice with cold PBS, and gently rocked on a shaker in 100 μl 1 × binding buffer. The binding buffer contained 2.5 μl of APC‐coupled Annexin V and 5.0 μl of 7‐AAD. The buffer was then incubated for 15 min in the dark at room temperature. Stained cells were analyzed using flow cytometry.^16^

### 2.9 Caspase activity assay

Caspase‐3 Activity Assay kit (Beyotime) was used to detect Caspase‐3 enzyme activity in cells by a colorimetric assay. Cells were washed twice with PBS and then lysed with 150 μl lysis buffer on ice for 20 min. After being centrifuged at 600 *g* at 4°C for 15 min, the supernatants were collected and quantified. Fifty microliters of supernatants (protein concentration reaches 1–3 mg/ml) were mixed with 40 μl buffer and 10 μl of Caspase reaction substrate (2 mM Ac‐DEVD‐pNA), incubated at 37°C for 1.5 h. The relative activity of Caspase was determined with a spectrophotometer (DU‐800, BECKMAN) at 405 nm.^11^

### 2.10 In vivo tumorigenic assays

BALB/C‐nu mice aged 4–6 weeks were purchased from the Shanghai SLAC Laboratory Animal Center of the Chinese Academy of Sciences. All animals were raised and maintained under pathogen‐free conditions. All experiments were approved by the Animal Conservation and Use Committee and were performed following institutional guidelines. Each group of cells (1 × 10^7^ cells in 200 μl PBS) was subcutaneously injected into the groin area of anesthetized nude mice (5 mice/group). Tumor volumes were recorded with digital calipers every 4 days, and the volume was calculated using the following formula: volume (mm^3^) = 0.5 × length × width^2^. At the termination of the experiment (Day 28), nude mice were euthanized using carbon dioxide, and the weight of the tumor was recorded. Each tumor was fixed in 4% paraformaldehyde for 48 h, embedded in paraffin, and sliced into 4 μm tissue sections for hematoxylin and eosin (H&E) and TUNEL staining.^17^, ^18^ A one‐step TUNEL apoptosis detection kit (KeyGen Biotech) was used to detect apoptotic cells in tumor tissues. In addition, 4′,6‐diamidino‐2‐phenylindole was used to stain nuclei. Digital images were captured using a fluorescence microscope (Nikon).

### 2.11 Gene expression profiling

A total of 2 × 10^5^ HeLa cells were inoculated into six‐well plates for 48 h. Each sample was repeated three times to extract RNA. PureLink^™^ RNA mini kit (Thermo Fisher Scientific) was used to separated total RNA based on instructions provided by the manufacturer. A Qubit® 3.0 Fluorometer (Life Technologies) was used to identify the quantity and quality of RNA samples to ensure that all samples RIN > 7.0. RNA samples were sent to Anoroad for library construction and sequencing. An Agilent 2100 RNA Library Prep Kit for Illumina (Agilent Technologies) was used to prepare the RNA library. Trim_galore (v0.4.2, Babraham Bioinformatics) was used to trim the low‐quality and cohesive sequences in the original reads, and RNA‐STAR (v2.4.0j) was used to map the clean reads to the reference genome (human genome 19). DEGSeq was used for genetic difference analysis, compared with the treatment group and the reference group and differential genes were selected with |log_2_Ratio| ≥ 1.5 and *q* < 0.05. KOBAS analysis of Gene Ontology (GO) and Kyoto Encyclopedia of Genes and Genomes (KEGG) for DE genes were used. The R Pathview software package was used to visualize the gene expression status in KEGG pathways.^19^

### 2.12 Statistical Analysis

All statistics were analyzed using SPSS 19.0 (SPSS Standard version 19.0, SPSS Inc.). A Student’s *t‐*test and one‐way analysis of variance were performed to compare different groups. Data were presented as the mean ± standard deviation (SD). All data were from at least three independent experiments. A *p* value of <0.05 was considered statistically significant, with the following notations: *p < 0.05, **p < 0.01.

## 3 RESULTS

### 3.1 Knockdown of NCL using shRNAs

**FIGURE 1:**
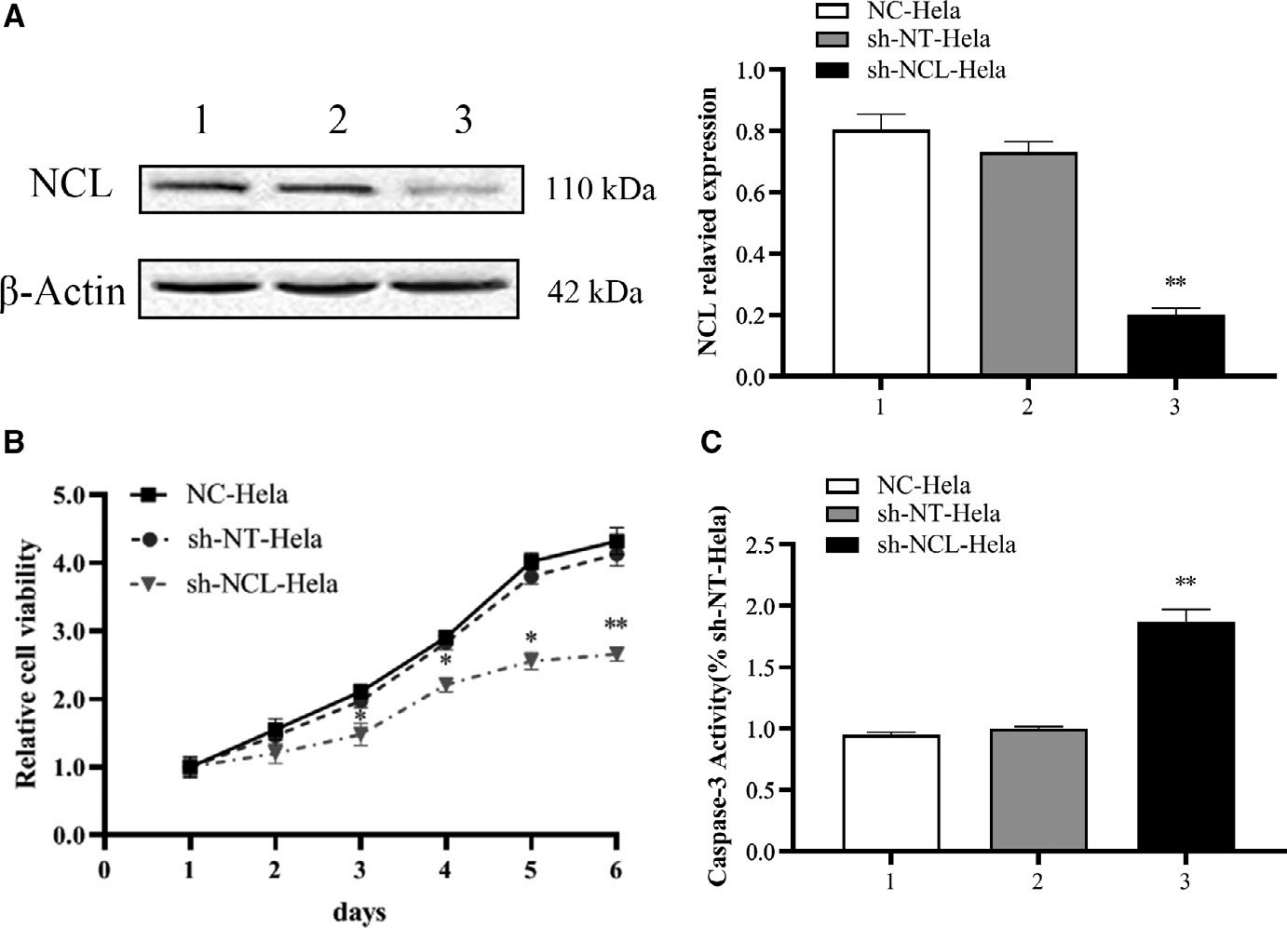
RNA interference inhibited NCL expression levels in HeLa cells. (A) NCL protein expression was detected by Western blotting. (B) Cell proliferation was detected by MTT analysis at 1, 2, 3, 4, 5, and 6 days. (C) Caspase‐3 activity. 1. NC‐HeLa, 2. sh‐NT‐HeLa, 3. sh‐NCL‐HeLa. Mean ± SD from three individual experiments. *p < 0.05, **p < 0.01, versus the sh‐NT‐HeLa group. MTT, 3‐(4, 5‐dimethylthiazol‐2‐yl)‐2, 5‐diphenyltetrazolium bromide; NCL, nucleolin

The efficiency of NCL knockdown in HeLa cells was verified by analyzing protein expression levels. Compared with sh‐NT‐HeLa cells, the NCLexpression level in sh‐NCL‐HeLa was reduced (**p < 0.01). Although there was no obvious difference observed between the sh‐NT‐HeLa and NC‐HeLa cells (Figure 1A), NCL was significantly downregulated in sh‐NCL‐HeLa cells.

### 3.2 NCL knockdown inhibits cell proliferation in vitro

**FIGURE 2:**
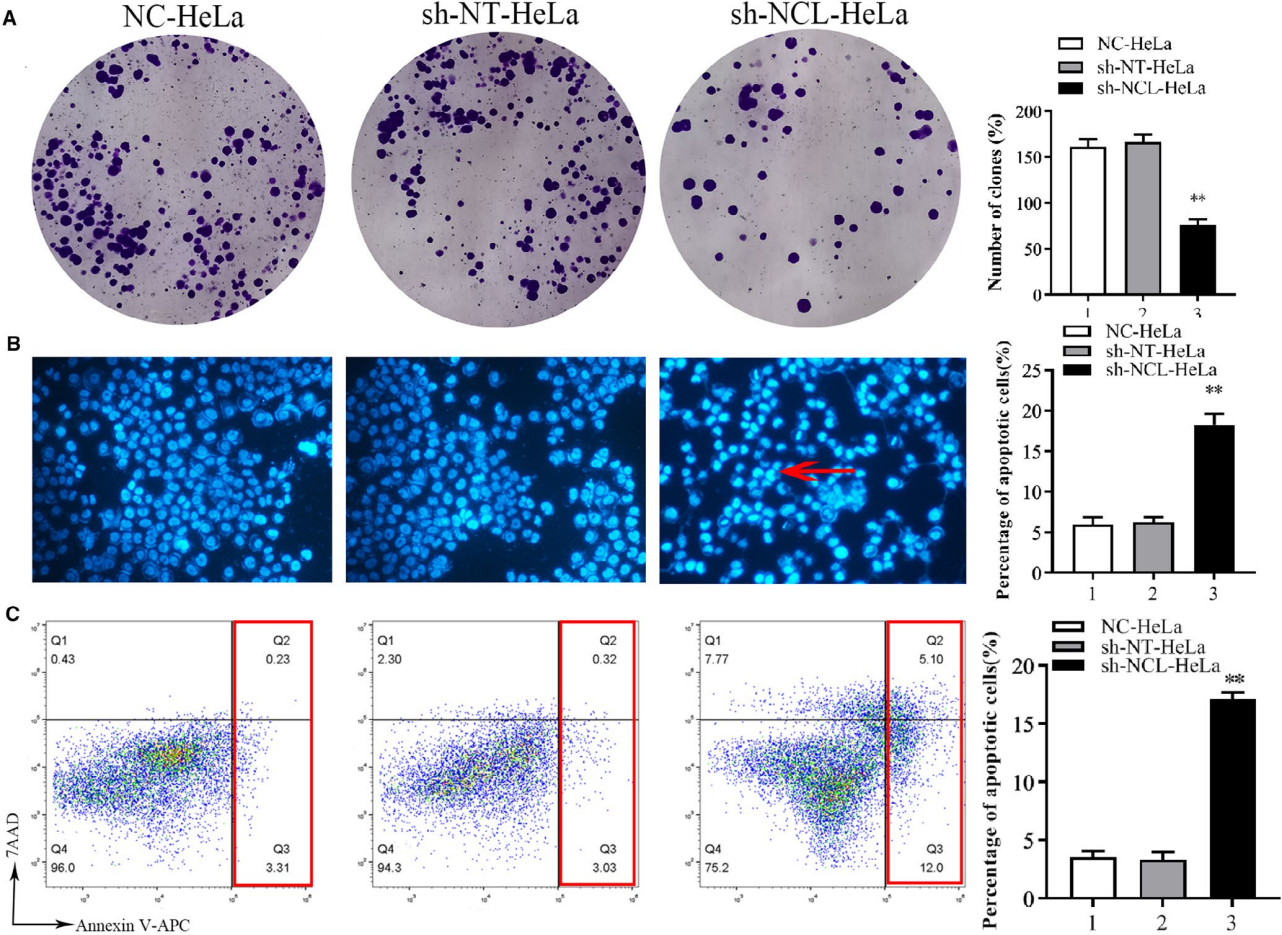
Detection of proliferation and apoptosis of HeLa cells after NCL interference. (A) Colony formation assay. Cells were inoculated into plates at a density of 500 cells per well and grown at 37°C for 14 days. Cell colonies were stained with 0.1% crystal violet (Left). Cell colonies were quantified, **p < 0.01 (right). (B) NCL knockdown induced apoptosis morphological features as shown by Hoechst. Hypercoagulable nuclei and semilunar apoptotic bodies represent typical apoptosis morphology (see the red arrow; fluorescence staining ×400). (C) Cell apoptosis was detected using flow cytometry. Representative flow cytometry analysis of Annexin V‐APC/7AAD staining (left). The apoptosis rate was quantified (right). Mean ± SD from three individual experiments. *p < 0.05, versus the sh‐NT‐HeLa group. NCL, nucleolin

Compared with sh‐NT‐HeLa cells, sh‐NCL‐HeLa proliferation was notably inhibited in a time‐dependent manner, and the highest inhibitory rate was 40.0 ± 1.7% on Day 6. However, there were no obvious differences observed in the sh‐NT‐HeLa and NC‐HeLa lines (Figure 1B). Colony formation assays revealed fewer and smaller colonies in sh‐NCL‐HeLa cells than in sh‐NT‐HeLa and NC‐HeLa cells (**p < 0.01) (Figure 2A). These results indicated that knockdown of NCL expression significantly inhibited the growth of HeLa cells in vitro.

### 3.3 NCL knockdown promotes apoptosis in HeLa cells

Hoechst 33342 staining was performed to identify how NCL knockdown regulated cell death. Results showed that there was a large number of hypercoagulable nuclei and semilunar apoptotic bodies in sh‐NCL‐HeLa cells (Figure 2B) and that apoptosis was more obvious at 48 h versus 24 h. Then we confirmed these findings using flow cytometry following Annexin V‐APC/7AAD staining. Consequently, a significant increase in apoptotic cells was observed in sh‐NCL‐HeLa cells (17.10 ± 0.71%), while low levels were observed in sh‐NT‐HeLa cells (3.35 ± 0.56%) and NC‐HeLa cells (3.54 ± 0.62%). There were significant differences noted between sh‐NCL‐HeLa cells and sh‐NT‐HeLa cells (**p < 0.01), no differences were observed between sh‐NT‐HeLa cells and NC‐HeLa cells (*p* > 0.05) (Figure 2C). Similarly, knockdown of NCL also increased Caspase‐3 enzyme activity (Figure 1C).

### 3.4 NCL knockdown significantly inhibited tumor formation of HeLa in vivo

**FIGURE 3:**
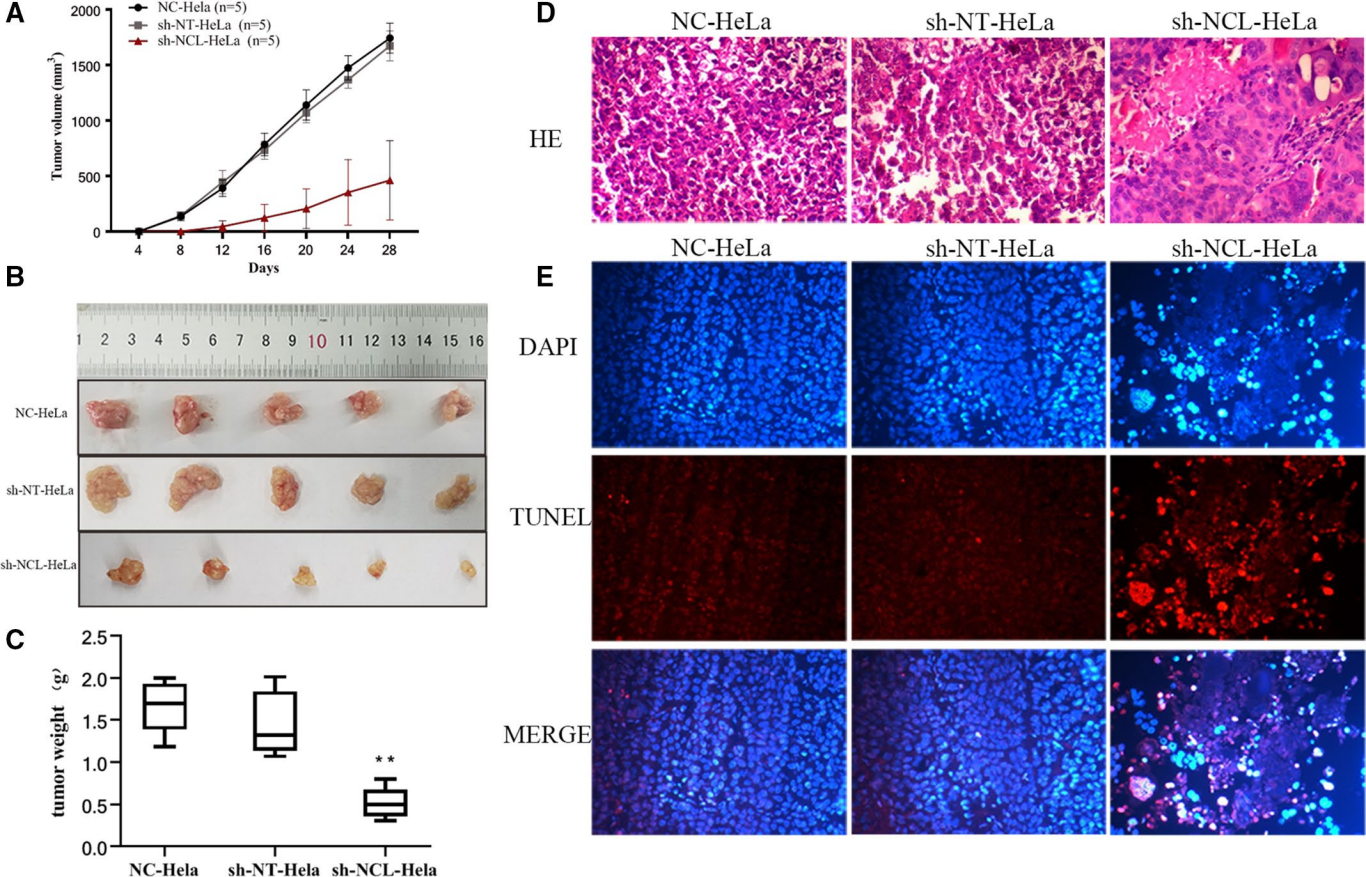
NCL knockdown regulates tumor formation in mice injected with HeLa cells. (A) Tumor volume was measured every 4 days, and the results were presented as a growth curve. (B) Representative images of tumors in nude mice after different treatments. (C) Tumor weight was measured on Day 28. (D) H&E staining (×200). (E) DAPI and TUNEL staining (×200). Results are presented as mean ± SD (*n* = 5). **p < 0.01, versus the sh‐NT‐HeLa group. DAPI, 4′,6‐diamidino‐2‐phenylindole; NCL, nucleolin

To examine the effects of NCL knockdown on in vivo tumor growth, HeLa cells were inoculated in nude mice and tumor volumes were measured up to 28 days. As shown in Figure 3, mice inoculated with sh‐NCL‐HeLa cells showed a significantly lower tumor growth curve (*p < 0.05). During the terminal period, the mean tumor volume in the sh‐NCL‐HeLa group was 0.38 times smaller than the sh‐NT‐HeLa group (626.9 ± 263.8 cm^3^ vs. 1614 ± 272.6 cm^3^, *p < 0.05), with an average weight being 0.513 ± 0.18 g for the sh‐NCL‐HeLa group and 1.454 ± 0.38 g for sh‐NT‐HeLa group (*p < 0.05). Using H&E staining, the degree of cell differentiation was higher in the sh‐NCL‐HeLa group compared with the control group, and the nucleus to cytoplasm ratio was also significantly reduced. Furthermore, TUNEL staining showed that the number of apoptosis‐positive cells in the sh‐NCL‐HeLa group (99.7 ± 12.6%) was significantly increased compared with the sh‐NT‐HeLa control group (0.9 ± 0.73%). Altogether, NCL knockdown in HeLa cells markedly suppressed in vivo tumor growth.

### 3.5 The PI3K/AKT pathway plays an important role in NCL‐regulated cancer cell proliferation and apoptosis

**FIGURE 4:**
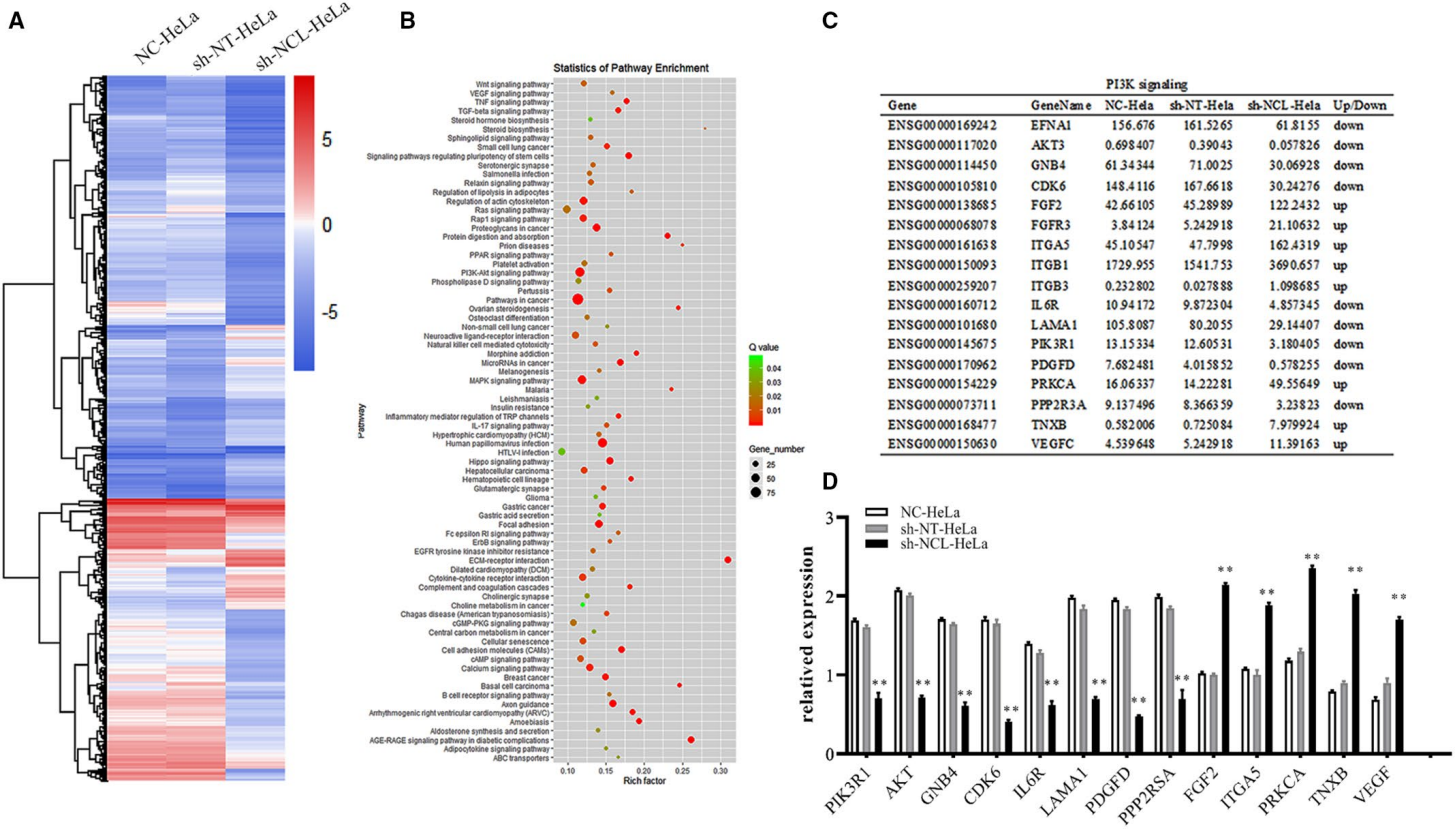
NCL knockdown induces apoptosis by inhibiting the PI3K/AKT pathway. (A) A heat map of differentially expressed genes in all samples. Rows represent genes, whereas columns represent samples. (B) Pathway enrichment analysis of transcriptome profiling. (C) Enrichment of differentially expressed genes in the PI3K/AKT pathway. (D) Diagram showing the fold‐change of several typical genes using RT‐PCR. **p < 0.01, versus the sh‐NT‐HeLa group. NCL, nucleolin

**FIGURE 5:**
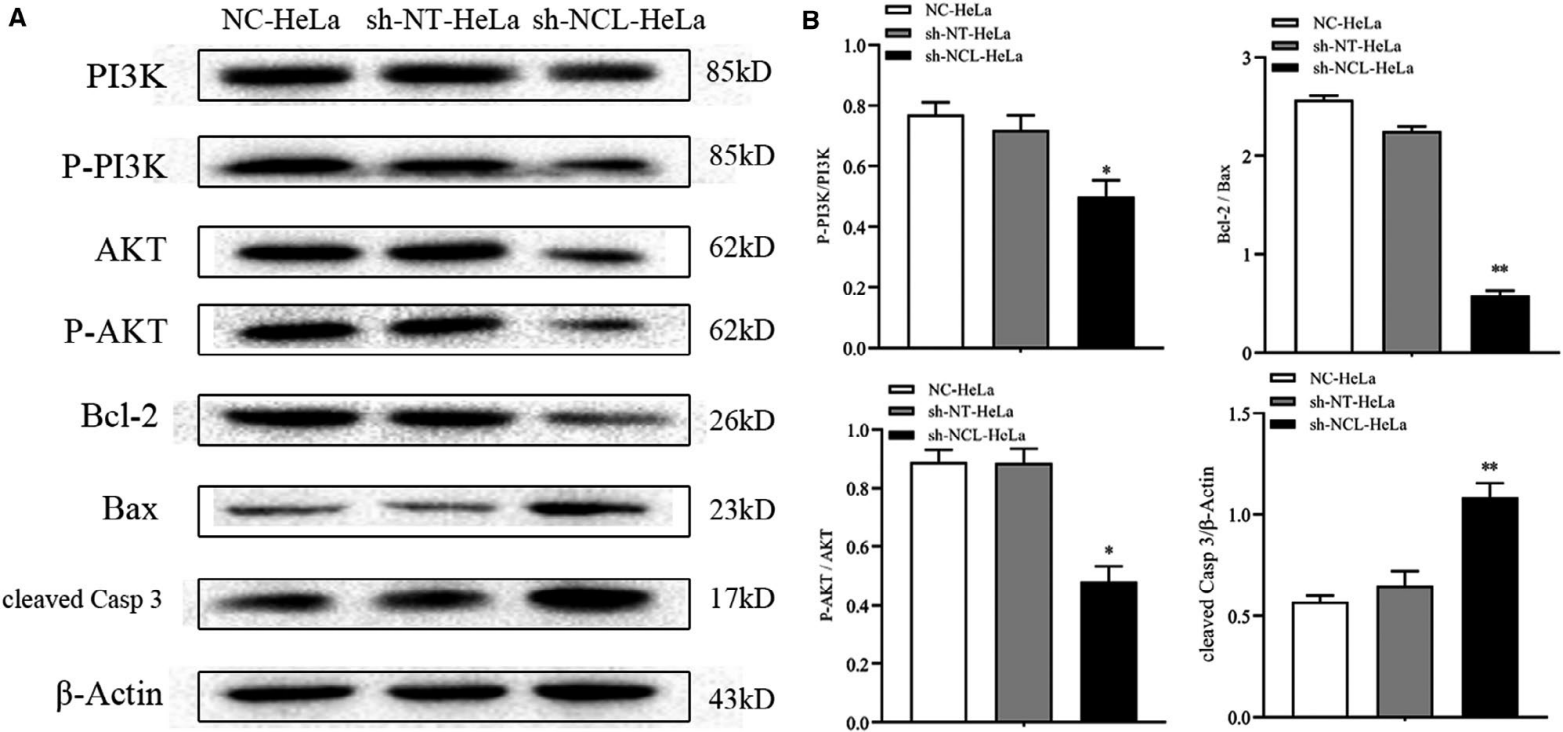
Protein levels of PI3K, AKT, Bcl‐2, Bax, and cleaved‐Caspase 3 were detected by Western blotting. (A) Representative Western blot brands. (B) Quantitative analyses of phospho‐PI3K/PI3K, phospho‐AKT/AKT, Bcl‐2/Bax, cleaved‐Caspase 3. Mean ± SD from three individual experiments. *p < 0.05, **p < 0.01, versus the sh‐NT‐HeLa group

Three groups of cells were collected, and RNA sequencing was performed to reveal mechanisms behind induced apoptosis. Heat map analysis showed that gene expression patterns differed between the sh‐NCL‐HeLa cells and the control cells (sh‐NT‐HeLa, NC‐HeLa, Figure 4A). A total of 2709 differentially expressed genes were identified using a fold‐change cutoff of >1.5 between sh‐NCL‐HeLa versus sh‐NT‐HeLa cells. A scatter plot of the common enrichment pathways was presented by KOBAS analysis (Figure 4B,C), which indicated that the number of genes in the PI3K/AKT pathway was enriched in the profiling data. In addition, the mRNA expression of several significantly altered genes was confirmed using RT‐PCR (Figure 4D). Western blotting revealed that there was a significant difference in protein expression levels between sh‐NT‐HeLa and sh‐NCL‐HeLa cells (*p < 0.01, Figure 5), the expression levels of phospho‐PI3K, phospho‐AKT, phospho‐PI3K/PI3K, phospho‐AKT/AKT, and Bcl‐2 in sh‐NCL‐HeLa cells significantly decreased, whereas the expression levels of cleaved‐Caspase 3 and Bax significantly increased.

### 3.6 Effects of NCL repression combined with PI3K pathway activation/inhibition on HeLa cell phenotypes

**FIGURE 6:**
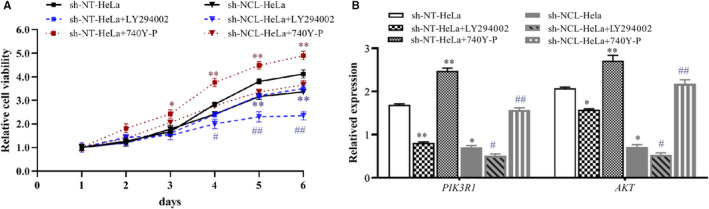
HeLa cells with repressed NCL levels were treated with the PI3K inhibitor LY294002 or the PI3K activator 740Y‐P. (A) Cell proliferation was determined using an MTT assay. (B) The expression levels of the apoptosis‐related genes were measured using RT‐PCR. *p < 0.05, **p < 0.01, versus the sh‐NT‐Hela group. ^#^ *p* < 0.05, ^##^ *p* < 0.01, versus the sh‐NCL‐Hela group, *n* = 3. MTT, 3‐(4, 5‐dimethylthiazol‐2‐yl)‐2, 5‐diphenyltetrazolium bromide; NCL, nucleolin

To confirm the association between NCL and the PI3K/AKT pathway, cells were treated with a PI3K inhibitor (LY294002) or a PI3K activator (740Y‐P). MTT results revealed that after treatment with LY294002, the cell proliferation in each group decreased, whereas the sh‐NCL‐HeLa group showed a significantly weaker proliferation rate compared with the sh‐NT‐HeLa group (*p < 0.05, ^#^ *p* < 0.05, Figure 6A). After treatment with 740Y‐P, cell proliferation was enhanced, with the sh‐NCL‐HeLa group showing significantly lower proliferation rates compared with the sh‐NT‐HeLa group (Figure 6A). In addition, RT‐PCR analyses revealed that mRNA expression levels of PI3K and AKT were lower after LY294002 treatment, with an opposite effect observed when cells were treated with 740Y‐P (Figure 6B). These results were consistent with results observed for sh‐NCL‐HeLa cells. Thus, NCL was found to play a role in inhibiting the proliferation and promoting apoptosis of HeLa cells through the PI3K/AKT pathway.

## 4 DISCUSSION

Studying NCL in malignant tumors began in the 1980s and its high expression was identified in different cancers such as liver cancer, prostate cancer, renal cell carcinoma, lung cancer, and colon cancer.^20^, ^21^, ^22^, ^23^, ^24^, ^25^ High expression of NCL promotes cell proliferation, angiogenesis, metastasis, and increased resistance to apoptosis in cancer cells. The distribution and structure of NCL in cells are closely related to pathological stage, tumor grade, increased disease risk, and patient survival rate.^26^ Our results showed that knockdown of NCL inhibited the proliferation and invasion of HeLa cells by promoting apoptosis. The tumor volume of nude mice inoculated with NCL knockdown cells was significantly smaller than that was observed in mice injected with normal and negative control group cells. In addition, tumor growth was significantly slower, indicating that NCL knockdown significantly inhibited cervical cancer growth, which was consistent with the previous findings observed in glioma.^27^ The results of pathological sections also revealed that downregulation of NCL expression induced apoptosis of transplanted tumor cells (Figure 3). Even though there is accumulating evidence of NCL pro‐tumor activity, data are still limited and the underlying molecular mechanisms remain unclear.

Transcriptomics allows us to interpret the functional elements of the genome and reveal overall gene expression profiles associated with tumors. This field has been used in cancer research and has led to an in‐depth understanding of tumorigenesis. The discovery of genes in the biological processes behind oncogenesis enhances the validation of potential diagnostic biomarkers. Ma et al. showed that UBC and NF‐κB were TLR9 target genes associated with HPV16 infection. TLR9 may activate UBC and NF‐κB through its signal transduction pathway and play a role in the occurrence and development of cervical cancer through the activation of NF‐kB downstream target genes.^28^ Li et al. transcriptome analysis using HeLa cells and HeLa/Dox cells to find 2562 differentially expressed genes. Moreover, many key genes in these pathways were expressed differently such as the PI3K/AKT pathway and MAPK pathway. In addition, most signaling pathways were linked to cell proliferation, signal transduction, transcription, and DNA repair.^29^ In this study, we showed that 40 classical signaling pathways were remarkably enriched, where the PI3K/AKT pathway was the most prominent. It is also important to note that PI3K, AKT, GNB4, CDK6, IL6R, LAMA, PDGFD, PPP2RSA, FGF, ITGA, PRKCA, TNXB, VEGF, and other genes in the pathway were all significantly altered.

Previous studies demonstrated that signal transmission in the PI3K/AKT pathway mainly relies on phosphorylation level changes by upstream key molecules to activate downstream protein factors. This pathway is then involved in transmitting signals from different cytokines to the nucleus and has a close and complex relationship with biological processes, such as cell survival, proliferation, apoptosis, invasion, and metastasis.^30^ Serine/threonine kinase AKT, the most important mediator, plays a major role in signal transduction throughout the PI3K pathway. Consequently, AKT was selected and tested for changes in transcription and expression levels regulated by NCL. This study revealed that the downregulation of NCL reduced AKT expression and inhibited PI3K/AKT pathway. Further data showed that GβY, Cytokine R, LAMA, and PDGFD, all of which are downstream genes of PI3K/AKT, were downregulated, whereas FGF, ITGA, TNXB, and VEGF genes were upregulated in the sh‐NCL‐HeLa cells, which exerted their effects on PRKCA and PKCs. Protein kinase Cα (PRKCA) is a serine/threonine protein kinase and a member of the PKC family. The PKC family regulates the ERK‐MAPK, PI3K/AKT, and NF‐κB pathways and is involved in a variety of cellular functions such as cell proliferation and survival.^31^ PRKCA is upregulated in several human cancer types including breast cancer, nonsmall‐cell lung cancer, and hematological malignancies.^32^, ^33^, ^34^ Protein phosphatase 2 regulatory subunit B alpha (PPP2R3A) regulates several important signal transduction pathways related to cancer including the Wnt signaling cascade, adenosine monophosphate‐activated protein kinase activity, and epidermal growth factor (EGF)/EGF receptor signaling pathway. PP2A is known to be involved in tumorigenesis and its function balances kinase‐mediated phosphorylation throughout cell signaling networks. Previous studies have shown that PP2A phosphorylates Akt and ERK. One report showed that treatment with OA, a PP2A inhibitor, not only inhibited PP2A activation but also triggered Akt and ERK signal transduction and increased cell growth, migration, and angiogenic ability. Thus, this supported the antiproliferation effects of PP2A in endothelial cells. Zhou et al. found that overexpression of Bcl‐2‐inhibited caspase‐3 activity, while caspase‐3 inactivation alleviated PP2A degradation, leading to a dissociation between PP2A and Akt.^35^ Caspase‐3 is the main executor caspase that can be cleaved and activated by caspase‐8, caspase‐9, and caspase‐10. Active caspase‐3 degrades a variety of cellular proteins and leads to morphological changes and DNA fragmentation during apoptosis.^36^ Mechanistically, our data from knockout studies indicated that the expression levels of active caspase‐3 were higher in sh‐NCL‐HeLa cells than in sh‐NT‐HeLa cells and were associated with apoptosis. At the same time, Akt phosphorylation of Bax changes it from being pro‐ to antiapoptotic, resulting in reduced mitochondrial sensitivity to apoptotic signal transduction. These reports both confirm and extend our results that the effects of NCL were related to the activation of PI3K/AKT pathways in HeLa cells. To assess the relationship between NCL and the PI3K/AKT pathway in cervical cancer, activators, and inhibitors of the PI3K/AKT pathway was used to observe changes in the two groups. The expression levels of apoptosis‐related genes in HeLa cells after using PI3K inhibitor LY294002 were consistent with sh‐NCL‐HeLa cells, while the effects of the agonist 740Y‐P were opposite. Altogether, these data indicate that decreased NCL expression may promote cell apoptosis by an independent mechanism in part by the PI3K/AKT pathway.

In conclusion, NCL plays an important role in the occurrence and development of cervical cancer and may be used as a biological marker for cervical cancer prevention and treatment. However, it still needs to be further verified by overexpression experiments, and more research is needed to elucidate the potential mechanism of other signaling pathways involved in the antiapoptotic effects of NCL and the changes of specific sites and domains in the PI3K/AKT signaling pathway.

## CONFLICT OF INTEREST

The authors declare that they have no conflict of interest.

## AUTHOR CONTRIBUTIONS

Jun Ying and Peizhen Li were responsible for the study design and critical revision of the manuscript. Zhouhao Tang, Jiayin Zhu, Ping Ren, and Yang Lou performed most of the experiments, Ruowang Pan analyzed the data, Jun Ying prepared all the figures and wrote the manuscript. Enyong Zhang, Dadao Huang, and Penghong Hu were responsible for the experiments in vivo. Dong Li and Qiyu Bao provided technical support. All authors have read and agreed to the published version of the manuscript.

## ETHICS APPROVAL AND CONSENT TO PARTICIPATE

All the experiments involving animals were reviewed and approved by the Ethics Committee of Wenzhou Medical University.

## Data Availability

All relevant data are within the article. The data that support the findings of this study are available from the corresponding author upon reasonable request.
